# Role of Concurrent Systemic Therapy with Adjuvant Radiation Therapy for Locally Advanced Cutaneous Head and Neck Squamous Cell Carcinoma

**DOI:** 10.7759/cureus.1784

**Published:** 2017-10-19

**Authors:** Uma Goyal, Nitin K Prabhakar, Rajayogesh Davuluri, Christopher M Morrison, Sun K Yi

**Affiliations:** 1 Radiation Oncology, University of Arizona Cancer Center; 2 College of Medicine, University of Arizona College of Medicine-Tucson

**Keywords:** cutaneous squamous cell carcinoma, adjuvant treatment, systemic therapy, postoperative radiotherapy

## Abstract

Objective

To evaluate the role of concurrent systemic therapy to postoperative radiation therapy (RT) for locally advanced cutaneous head and neck squamous cell carcinoma (LA-cHNSCC).

Materials and methods

A retrospective study of 32 patients with LA-cHNSCC receiving postoperative RT with and without systemic therapy was conducted. Patients with LA-cHNSCC after surgical resection with one or more high risk features were evaluated. Local regional control (LRC), distant control (DC), and acute and late toxicities were evaluated with Fisher exact tests. Progression-free survival (PFS) and overall survival (OS) were evaluated utilizing Kaplan Meier and log-rank analyses. Univariate Cox proportional hazard analyses were used to examine patient, disease, and treatment-related factors with OS and PFS.

Results

While comparing patients receiving RT with systemic therapy (n = 14) vs RT alone (n = 18), LRC was 92.9% vs 72.2% (p = 0.20), DC 92.9% vs 94.4% (p = 1.0), median PFS 17.7 months vs 34.4 months (p = 0.48), and median OS 20.9 months vs 34.4 months (p = 0.03), respectively. On univariate analyses, use of concurrent systemic therapy was associated with an increased risk of death with an HR of 3.5 [95% confidence interval (CI): 1.04 - 11.6] (p = 0.04), while patients treated for recurrent disease who had previously treated superficial primaries had improved OS with an HR of 0.10 [95% CI: 0.01-0.80] (p = 0.03). There were no significant differences in acute or chronic toxicities between groups.

Conclusions

Patients receiving postoperative RT alone for LA-cHNSCC had better OS than patients receiving concurrent systemic therapy. There were no differences in any other endpoints evaluated.

## Introduction

Non-melanoma skin cancers are the most commonly diagnosed malignancy in the United States with an estimated 3.5 million cases reported in 2006 [1], and approximately 20% of those are squamous cell in origin [2]. The lifetime cumulative risk for developing cutaneous squamous cell carcinoma (cSCC) is estimated to range between 5-15%, with men having a higher predisposition than women [3]. Approximately 60% of cSCC originate in the head and neck (HN) region [4], and longitudinal studies from North America have reported a sharp increase in the incidence of cSCC over the last several decades with current trends indicating up to a threefold increase in the number of cases diagnosed [5]. These findings are striking and suggest an emerging public health concern.

Unlike the management for early stage and superficial cSCC, which is often highly curable and includes a variety of single modality options [6], the management of locally advanced cutaneous head and neck squamous cell carcinoma (LA-cHNSCC) is not clearly defined. Treatment for these patients often includes surgical resection, followed by adjuvant therapy based on small retrospective studies previously published [7]. Many disease-related factors have been strongly correlated with higher rates of failure for cSCC and include primary lesions with increasing size, depth of invasion, location near sensitive structures like the eyes, ears, nose, and mouth, poorer histologic differentiation, recurrence after prior treatment, host immunosuppression, and the existence of perineural or lymphovascular invasion. The local and metastatic recurrence rates associated with these high risk features range between 15-50% [8]. Patients found with positive margins, extensive perineural invasion, or large nerve involvement have been shown to harbor the greatest risk of recurrence [9]. Additionally, drainage of LA-cHNSCC, which is known to occur within intra- and periparotid lymph nodes as well as upper cervical nodal regions, has also been associated with poorer outcomes [10]. Patients therefore found with any of these high risk features upon surgical resection are often referred for postoperative radiotherapy (RT).

Studies of locally advanced mucosal head and neck squamous cell carcinoma (LA-mHNSCC) have shown that the addition of cisplatin-based concurrent chemotherapy with postoperative RT improves disease outcomes over postoperative RT alone, with the greatest benefit detected in patients found with extracapsular nodal extension or positive surgical margins [11-13]. At the present time, there is a lack of randomized data to help guide practitioners treating LA-cHNSCC postoperatively in patients who have a high risk of recurrence, though prospective studies evaluating the role of concurrent systemic therapy with postoperative RT for these patients are currently accruing. The objective of this study was to determine the impact in disease-related outcomes and toxicity when treating LA-cHNSCC with postoperative RT with or without concurrent systemic therapy.

## Materials and methods

Patient characteristics

This study included 32 patients treated at our institution from 2007-2016, who had received postoperative RT with or without concurrent systemic therapy as part of their management for LA-cHNSCC. We received approval from our institutional review board (#1605586135​) prior to initiation of this retrospective study. Patients, aged 18 or older, who were diagnosed with LA-cHNSCC and underwent definitive surgical resection followed by RT were included in this study. Patients were considered locally advanced if they had recurrent disease, nodal involvement, a bulky or invasive primary, perineural invasion, or gross/microscopic positive margins. Patients were staged according to tumor/node/metastases (TNM) spread based on the American Joint Committee on Cancer (AJCC) version 7 staging handbook [14].

Radiation therapy

All patients were treated with RT using an aquaplast HN mask for immobilization (Thermoplastics, Civco Radiotherapy, Coralville, IA). A computed tomography (CT) simulation scan was performed with 3 mm thick slices through the affected HN area (Philips CT Big Bore CT Simulator, USA) for treatment planning. A fusion of preoperative and postoperative imaging to help delineate the tumor bed and normal structures was done when applicable. RT was delivered once or twice daily (fractions at least six hours apart if delivered twice daily) consecutively on Mondays through Fridays. RT breaks were considered as any missed fraction during the planned course of treatment, regardless of length or reason for cancellation.

Systemic therapy

Fourteen patients received concurrent systemic therapy with their postoperative RT. Five patients were treated with concurrent cisplatin-based monotherapy. Four patients were treated with concurrent carboplatin-based monotherapy. Two patients were treated with concurrent cetuximab-based monotherapy. Two patients were treated with concurrent carbotaxol-based doublet chemotherapy and one patient was treated with concurrent cisplatin + 5-fluorouracil-based doublet chemotherapy. One of two patients who were immunosuppressed received systemic therapy with cisplatin.

Statistics

The primary endpoint of the study was whether the addition of concurrent systemic therapy to postoperative RT would improve locoregional control (LRC) in LA-cHNSCC patients. Secondary endpoints evaluated were: distant control (DC), progression free survival (PFS), overall survival (OS), acute toxicities, and late toxicities. Acute toxicities were defined as those treatment-related sequelae occurring during or within three months of the end of RT. Late toxicities were defined as those occurring more than three months post RT. Comparison of continuous variables was conducted with unpaired, two-tailed Student’s t-tests. The differences in the frequency of occurrences including LRC, DC, and toxicities between the two groups were analyzed using Fisher’s exact test. PFS and OS were evaluated with Kaplan-Meier (K-M) projections and the differences between groups were analyzed using log-rank tests. Univariate Cox proportional hazard analyses were used to examine the association of patient, disease, and treatment related factors with OS and PFS. Multivariate analyses were unable to be performed given insufficient data. Differences between groups were considered statistically significant when p<0.05. Analyses were performed using version 3.3.1 of the open-source software package R (www.r-project.org).

## Results

Patient and disease characteristics

A total of 32 patients were included in this study. Fourteen patients received postoperative RT with concurrent systemic therapy and 18 patients received postoperative RT alone. The median age of patients for RT with systemic therapy was 71 years and RT alone 74 years (p=0.26). Two patients were immunosuppressed with one in each group. Two patients in each group had >T2 stage (p = 1.0). Seven patients who received RT with systemic therapy and four patients with RT alone had >N1 stage (p = 0.14). Thirteen patients had recurrent disease from initially treated superficial primary lesions, four of which received systemic therapy. All patients were found with a Karnofsky Performance Status (KPS) of ≥ 70 when known. Table 1 summarizes the patient and disease characteristics.

**Table 1:** Patient and disease characteristics KPS = Karnofsky performance score.

Patient Characteristics	RT+systemic therapy (n = 14)	RT alone (n = 18)
Median age (yrs)	71	74
Male (n)	14	18
KPS (n)		
70	1	0
80	7	2
90	6	12
Unknown	0	4
Immunosuppressed (n)	1	1
Disease Characteristics	RT+systemic therapy (n = 14)	RT alone (n = 18)
T Stage		
1	1	2
2	6	9
3	0	2
4	2	0
Unknown	5	5
N stage		
0	4	8
1	2	4
2	7	4
Unknown	1	2
Recurrent lesion	8	12
Originally Superficial	4	7
Perineural invasion	4	9
Lymphovascular invasion	4	3
Positive margin	6	5
Extranodal Extension	6	2
Differentiation		
Well	4	4
Moderately	2	5
Poorly	3	6
Unknown	5	3

Treatment characteristics

The overall median follow-up was 30.1 months. The median follow-up time for the RT with systemic therapy group was 20.9 months and RT alone was 34.4 months (p=0.045). Of the patients who received concurrent systemic therapy, five patients received single agent cisplatin at doses ranging from 70-100 mg/m2 in 1-3 cycles. One patient completed three cycles of cisplatin at 100 mg/m2. Two patients completed two cycles of cisplatin at 100 mg/m2. One patient completed two cycles of cisplatin at 100 mg/m2 followed by a dose reduced cycle at 70 mg/m2. The final patient completed one cycle of cisplatin at 70 mg/m2. Four patients received between 2-7 cycles of weekly single agent carboplatin using area under curve (AUC) dosing. Two patients completed seven cycles of carboplatin, one patient completed six cycles, and one patient completed two cycles. Two patients received concurrent cetuximab monotherapy, with one completing a loading dose of 400 mg/m2 followed by six weekly cycles of 250 mg/m2 and another completing a loading dose of 400 mg/m2 followed by two weekly cycles of 250 mg/m2, followed by a final dose reduced cycle at 100 mg/m2. Two patients each completed two cycles of carboplatin and paclitaxel, and one patient completed two cycles of 20 mg/m2 of cisplatin and 1000 mg/m2 of 5-fluoruracil every three weeks.

Total RT doses delivered ranged from 37.8-70 gray (Gy) in 1.8-3 Gy daily fractions or 1.25-1.5 Gy twice daily fractions. The average duration of treatment for all patients was 46.3 total elapsed days, and there was not a significant difference between RT with systemic therapy vs RT alone groups (45.6 elapsed days vs 46.7 elapsed days, respectively; p = 0.80). The range of RT duration spanned from 29-62 elapsed days for the RT with systemic therapy group and 22-92 elapsed days for the RT alone group. Twelve (85.7%) of the patients receiving RT with systemic therapy required at least one RT break from their planned RT schedule versus 11 (61.1%) of the patients receiving RT alone (p = 0.23). Among those patients who required RT breaks, the average duration of the RT breaks did not significantly differ between the two groups (3.5 days for RT with systemic therapy vs 6.7 days for RT alone; p = 0.18).

Outcomes

**Figure 1:**
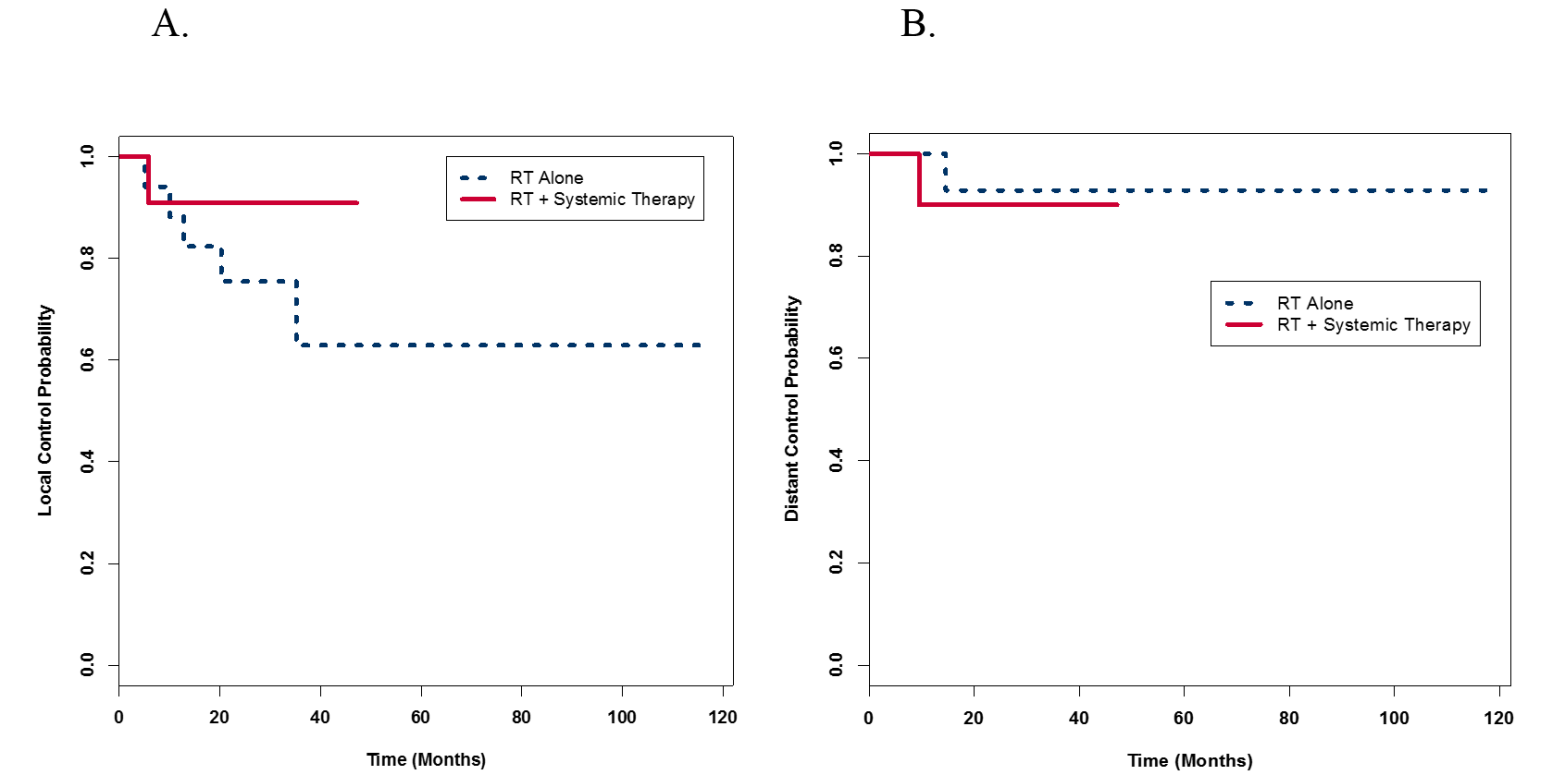
Locoregional and distant control probability Kaplan Meier curves showing locoregional (A) and distant (B) control probabilities with no significant differences between the radiation therapy (RT) with systemic group versus RT alone groups (p = 0.30 and 0.71, respectively).

LRC for RT with systemic therapy was 92.9% and RT alone was 72.2% (p = 0.20). The DC for RT with systemic therapy was 92.9% and RT alone was 94.4% (p = 1.0). Figure 1A provides K-M probability curves for LRC and Figure 1B for DC. There were no statistically significant differences between groups with respect to LRC or DC.

**Figure 2:**
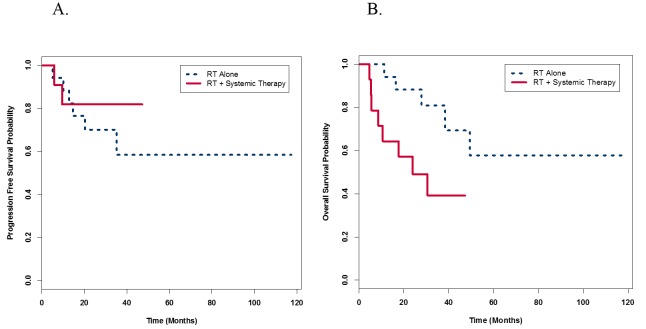
Progression free and overall survival probability (A) Progression free survival Kaplan Meier curves show non-significant differences between radiation therapy (RT) with systemic therapy compared to RT alone groups (p = 0.48). (B) Kaplan Meier curves for overall survival (OS) shows significantly decreased OS in the RT with systemic therapy group compared to RT alone (p = 0.03).

The median PFS for the RT with systemic therapy group was 17.7 months vs 34.4 months for the RT alone group (Figure 2A). There was no statistically significant difference in PFS between the two groups (p = 0.48). The median OS for RT with systemic therapy was 20.9 months vs 34.4 months for RT alone (Figure 2B). In contrast to PFS, there was a statistically significant difference in OS favoring the RT alone group (p = 0.03).

On univariate analysis, the use of concurrent systemic therapy was associated with a significant decrease in OS with an HR of 3.5 [95% confidence interval (CI): 1.04 - 11.6] (p = 0.04). The use of systemic therapy, however, did not have a significant influence on PFS. Treated disease that was found as recurrences from initially treated superficial primaries had significantly improved OS with an HR of 0.10 [95% CI: 0.01-0.80] (p = 0.03). None of the other patient, disease, or treatment characteristics examined had significant influences on PFS or OS on univariate analyses. The full results of those analyses are displayed in Table 2.

**Table 2:** Univariate analysis PFS = progression free survival; OS = overall survival; LVSI = lymphovascular space invasion; PNI = perineural invasion.

PFS	HR	95% CI	p-value	OS	HR	95% CI	p-value
Systemic Therapy	0.57	0.11 - 2.8	0.49	Systemic Therapy	3.5	1.04 - 11.6	0.04
LVSI	2.7	0.54-13.58	0.22	LVSI	1.26	0.38-4.11	0.71
PNI	7.1	0.83-61.15	0.07	PNI	0.88	0.29-2.71	0.83
Positive margin	1.77	0.36-8.83	0.49	Positive margin	1.44	0.48-4.30	0.52
Superficial Recurrence	0.3	0.03-2.58	0.27	Superficial Recurrence	0.1	0.01-0.80	0.03
Age	1.03	0.95-1.12	0.49	Age	1	0.96-1.06	0.79
N0 stage	0.81	0.08-8.32	0.86	N0 stage	0.44	0.07-2.72	0.38
N1 stage	0.47	0.03-7.81	0.6	N1 stage	0.72	0.12-4.43	0.73
N2 stage	0.27	0.02-4.40	0.36	N2 stage	0.7	0.14-3.63	0.67

Toxicity

There were no significant differences between groups for any grade acute or late toxicities (Table 3).

**Table 3:** Acute and late toxicities

Acute toxicities			
Toxicity	RT+ systemic therapy (n)	RT alone (n)	p-value
All dermatologic	4	8	0.47
Diarrhea	1	0	0.48
Dysgeusia	3	4	1
Dysphagia	3	0	0.098
Fatigue	3	0	0.098
Nausea	1	0	0.48
Odynophagia	1	2	1
Oral mucositis	4	2	0.38
Tinnitis	0	2	0.48
Weight loss	2	1	0.33
Xerostomia	5	4	0.69
Late toxicities			
Toxicity	RT+systemic therapy (n)	RT alone (n)	p-value
Soft tissue necrosis	0	1	1
Dysgeusia	1	0	0.48
Desquamation	0	1	1
Depression	1	0	0.48

## Discussion

To better define the optimal management for LA-cHNSCC, this retrospective study examined the role of postoperative RT with concurrent systemic therapy, including platinum-based chemotherapy or cetuximab, compared to postoperative RT alone. The management of LA-cHNSCC is currently not clearly defined.

Two large prospective randomized cooperative group clinical trials published in 2004 helped define the optimal postoperative management of LA-mHNSCC [11-12]. In a subsequent pooled meta-analysis of these two studies, it was determined that patients with positive margins or extracapsular nodal extension benefited most with an OS advantage when receiving concurrent cisplatin during their postoperative RT [13]. NCCN guidelines currently recommend an extrapolation from the mSCC literature when deciding upon the optimal adjuvant therapy for LA-cHNSCC [15]. The decision to offer patients postoperative RT vs postoperative RT with systemic therapy is therefore often done by institutional preference [7]. Though no standard postoperative RT systemic therapy regimen exists at the present time, a number of prospective trials are currently accruing to evaluate platinum or epidermal growth factor receptor (EGFR) inhibitor-based regimens based on prior responses seen in the cSCC literature [16-17].

Our study found no difference in LRC, DC, or PFS between patients who received postoperative RT with or without concurrent systemic therapy when treated adjuvantly for LA-cHNSCC. There was a difference in OS, however, which favored the group receiving RT alone. There are multiple possible explanations for this finding. The first is that the use of systemic therapy contributed to increased deaths in those who received combined modality treatment versus RT alone. The lack of differences in baseline KPS or in the development of treatment related toxicities between groups, however might suggest otherwise. Unfortunately, we were not able to calculate comorbidity indices for our patients, which could have produced more meaningful differences on the survival impact of systemic therapy for this patient population. Second, given the retrospective nature of this analysis, there are likely other confounding factors, such as selection bias, which have been left unaccounted for. Despite lack of obvious differences in patient and disease characteristics between groups, there may have been a greater proportion of patients with higher risk disease that were selected for RT with systemic therapy, which may have been responsible for poorer outcomes in this group despite more aggressive treatment. Third, though there were no differences in the number of RT breaks or total RT days missed during breaks between groups, which is a known negative predictor for disease outcomes [18], patients in the combined modality group may have been delivered suboptimal systemic therapy.

Among the 14 patients who received systemic therapy in our study, three had clearly suboptimal delivery of systemic therapy. There are data that suggest a direct correlation between the cumulative dose of concurrent cisplatin received and improved patient outcomes [19], although in the postoperative trial of concurrent cisplatin by Bernier, et al. [11], for LA-mHNSCC, approximately 20% of patients that received combined modality treatment failed to complete all courses of their planned chemotherapy. Given the heterogeneity of systemic therapy delivered in our study, as demonstrated by the wide range of agents, dosing, and schemas utilized, it is difficult to estimate what impact suboptimal delivery of systemic therapy might have had on the poorer outcomes in the combined modality group. Lastly and importantly, the significantly longer follow-up time for the RT alone group, a variable that directly impacts the calculation of median OS, could have impacted our OS results.

On univariate analyses for OS, we found two variables that were statistically significant. In addition to discovering that patients who received systemic therapy had greater risk of death than those that did not, as described above, we also found that patients with LA-cHNSCC who were treated as a recurrence from a prior initially treated superficial primary tumor had significantly better outcomes than patients presenting with other aggressive features. This finding may have been confounded by the fact that there was a trend for more of these patients to have received RT alone.

The limitations of this study are those commonly seen with any retrospective study, but also include the small number of patients, wide ranging doses of postoperative RT, heterogeneity of systemic therapy agents and dosing, and variable length in follow-up. As a result, it is difficult to draw definitive conclusions about the role of concurrent systemic therapy with postoperative RT for LA-cHNSCC based upon this study alone. However, given the paucity of available studies on this topic, our institutional experience adds some value to the scant literature. We anxiously await the results of currently ongoing prospective studies to help direct optimal care for this particular patient population.

## Conclusions

Patients receiving systemic therapy with postoperative RT appeared to have worse OS outcomes than those receiving RT alone in our small sample of patients, which may be attributable to selection bias where higher risk patients received systemic therapy, but could have also reflected suboptimal delivery of systemic therapy, or an increased risk of death in patients receiving combined treatment. Results of larger prospective randomized studies are needed to draw firmer conclusions on the impact of concurrent systemic therapy with adjuvant RT for LA-cHNSCC.
